# Bacterial Inhibition and Osteogenic Potentials of Sr/Zn Co-Doped Nano-Hydroxyapatite-PLGA Composite Scaffold for Bone Tissue Engineering Applications

**DOI:** 10.3390/polym15061370

**Published:** 2023-03-09

**Authors:** Mozan Hassan, Abbas Khaleel, Sherif Mohamed Karam, Ali Hassan Al-Marzouqi, Ihtesham ur Rehman, Sahar Mohsin

**Affiliations:** 1 Department of Anatomy, College of Medicine and Health Sciences, United Arab Emirates University, Al Ain P.O. Box 15551, United Arab Emirates; 2 Department of Chemistry, College of Science, United Arab Emirates University, Al Ain P.O. Box 15551, United Arab Emirates; 3 Department of Chemical and Petroleum Engineering, College of Engineering, United Arab Emirates University, Al Ain P.O. Box 15551, United Arab Emirates; 4 School of Medicine, University of Central Lancashire, Preston PR1 2HE, UK

**Keywords:** strontium, zinc, nano-hydroxyapatite, PLGA, antibacterial, bone scaffolds, cell proliferation

## Abstract

Bacterial infection associated with bone grafts is one of the major challenges that can lead to implant failure. Treatment of these infections is a costly endeavor; therefore, an ideal bone scaffold should merge both biocompatibility and antibacterial activity. Antibiotic-impregnated scaffolds may prevent bacterial colonization but exacerbate the global antibiotic resistance problem. Recent approaches combined scaffolds with metal ions that have antimicrobial properties. In our study, a unique strontium/zinc (Sr/Zn) co-doped nanohydroxyapatite (nHAp) and Poly (lactic-co-glycolic acid) -(PLGA) composite scaffold was fabricated using a chemical precipitation method with different ratios of Sr/Zn ions (1%, 2.5%, and 4%). The scaffolds’ antibacterial activity against *Staphylococcus aureus* were evaluated by counting bacterial colony-forming unit (CFU) numbers after direct contact with the scaffolds. The results showed a dose-dependent reduction in CFU numbers as the Zn concentration increased, with 4% Zn showing the best antibacterial properties of all the Zn-containing scaffolds. PLGA incorporation in Sr/Zn-nHAp did not affect the Zn antibacterial activity and the 4% Sr/Zn-nHAp-PLGA scaffold showed a 99.7% bacterial growth inhibition. MTT (3-(4,5-Dimethylthiazol-2-yl)-2,5-diphenyltetrazolium bromide) cell viability assay showed that Sr/Zn co-doping supported osteoblast cell proliferation with no apparent cytotoxicity and the highest doping percentage in the 4% Sr/Zn-nHAp-PLGA was found to be ideal for cell growth. In conclusion, these findings demonstrate the potential for a 4% Sr/Zn-nHAp-PLGA scaffold with enhanced antibacterial activity and cytocompatibility as a suitable candidate for bone regeneration.

## 1. Introduction

During a lifetime, skeletal bones are subjected to massive physical stress that can induce bone remodeling and affect bone function and structure [1]. To withstand this physical stress, the bone is a highly dynamic organ composed of ~60% inorganic matter, ~30% organic matter, and ~10% water [2]. The inorganic phase is represented mainly by calcium phosphate present in the form of hydroxyapatite (HA) crystals permeated through the gaps of the parallelly oriented type I collagen fibers which represent the organic extracellular matrix. HA is responsible for bone stiffness, while collagen provides elasticity and tensile strength [3].

Bone fractures have been imitatively restored using autogenous bone grafts which can avoid immune rejection but have shown numerous drawbacks such as pain, long recovery periods, infections, and limited quantity. Another possible treatment for bone defects is allogenic grafts and xenografts, but their use has been limited due to biological issues such as immune rejection and post-implantation infections, not to mention ethical issues [4]. Nowadays, the whole globe is facing a growing demand for bone grafts due to the increase in the numbers of the elderly population, with over 2 million bone replacement surgeries performed per annum [5]. 

Bone tissue engineering (BTE) using synthetic bone substitutes has been investigated extensively as a promising approach for the treatment of bone disorders. The approach is anticipated to fully supersede the currently used bone grafts in clinical applications and augment bone repair and regeneration [6]. To achieve this, the grafted biomaterial should fulfill certain criteria such as (i) biocompatibility, (ii) biodegradability that is tailored to the new bone formation, (iii) the provision of proper mechanical support similar to the injured bone, (iv) the enablement of cell attachment and vascularization, and (v) the promotion of osteogenesis and allowance of osteointegration [7,8,9,10]. Biomaterials of different origins have been used to bridge the gap and restore the strength of weak, broken, or deficient bones. These include natural polymers [11], synthetic polymers [12], and ceramics [13]. 

Synthetic calcium phosphate scaffolds, mainly in form of HA, are widely used in BTE due to their osteoconductivity and bioactivity. HA is a natural bone component known to enhance cell attachment due to its ability to adsorb more cell-adhesive plasma proteins, such as fibronectin and vitronectin, resulting in more adherent cells [14]. However, its sole use has been limited due to its poor mechanical properties [15]. Recent technologies have adopted synthetic calcium-phosphate-dependent scaffolds that are loaded with biological compounds such as growth factors or drugs to enhance bone formation. The main challenge in these scaffolds includes drug/growth factor low solubility, effective dose assessment, short half-life, and side effects such as ectopic bone formation [16]. A prospective safer approach that has recently been investigated is the addition of natural bone trace elements such as magnesium, manganese, zinc, and strontium ions (Mg^2+^, Mn^2+^, Zn^2+^, and Sr^2+^) that can naturally induce growth factor production by cells to promote osteogenesis [17]. Additionally, these elements can improve scaffold biological responses and physical properties [18]. 

Zinc (Zn) is the most abundant ion in bones and is widely used to substitute Ca^2+^ in HA [19]. Zn^2+^ is also known to play a key role in the immune system and participate in numerous anabolic and catabolic activities that help maintain cellular integrity. In addition, Zn plays an essential role in transcription and gene expression pathways [20]. Many studies demonstrated that zinc could promote bone formation and enhance the expression of osteoblastic gene markers, as well as inhibit osteoclast bone resorption, although the full pathway remains to be disclosed [21]. Yamaguchi et al. proposed that zinc can potentially act as an NF-κB (nuclear factor kappa light chain enhancer of activated B cells) pathway antagonist in both osteoblasts and osteoclasts, decreasing both bone resorption and formation; the negative effect on bone formation will later be modulated by activating the Smad (Suppressor of Mothers against Decapentaplegic) pathway that plays a critical role in osteoblastic lineage commitment and proliferation, hence increasing bone mineralization [22]. Furthermore, Grandjean-Laquerriere et al. showed that zinc can increase the production of anti-inflammatory cytokines such as interleukin (IL) IL-10 and IL-8, simultaneously decreasing the production of inflammatory cytokines such as TNF-α (tumor necrosis factor alpha) [23]. In addition, Zn^2+^ is also known to have antibacterial properties that have been reported in many studies [24,25,26,27].

Zn’s antibacterial property is very important as bacterial growth at the implantation site is one of the major failures in the bone grafting and healing process. The infection is mainly caused by bacterial strains that can form a biofilm and produce an extracellular polymeric layer that can help bacteria to survive and escape the immune system, as well as antimicrobial agents [28]. A total of 75% of post-implant infections are caused by the pathogen *Staphylococcus aureus* (*S. aureus*) which can colonize asymptomatically, resulting in a life-threatening disease and implant failure [29]. To overcome the post-implantation bacterial infection, recent studies have investigated physical antibacterial mechanisms using surface coats that can inhibit bacterial adhesion and growth, such as nanopillars which can stick to the bacterial cell membrane, rupturing it and eventually preventing biofilm formation [30]. However, the use of this method has been limited due to the high temperature, pressure, and electrical energy techniques that are adopted in the synthesis of these surfaces which can interfere with polymers’ physical properties [31]. In contrast, several studies investigated combining the scaffold with chemical antimicrobial agents such as antibiotics and metal ions (e.g., zinc, silver, and copper) [32], with zinc ions showing preferable cell differentiation and antimicrobial properties [33].

Despite all of zinc’sadvantages, many studies showed that higher Zn^2+^ concentrations are cytotoxic to cells and can lead to the accumulation of zinc ions in mitochondria leading to functional impairment and cell apoptosis [34]. Guo et al. showed that cells exposed to 10 µg/mL of zinc oxide for 4 h can reduce cell viability to less than 30% [35]. In addition, Wang et al. reported that higher Zn^2+^ concentrations of more than 5% showed a cytotoxic effect on human bone marrow mesenchymal stem cell (hBMSCs) cultures, with 10% Zn showing reduced alkaline phosphatase (ALP) activity compared to lower Zn concentrations [36]. 

Strontium (Sr) is a divalent cation that has chemical properties analogous to calcium and can be processed by the body in a similar way to calcium [37]. Sr has a dual effect on both osteoblasts and osteoclasts. In osteoblasts, Sr^2+^ binds to Ca^2+^ receptors and enhances cell proliferation and bone formation. Concurrently, in osteoclasts, strontium ions (Sr^2+)^ can induce conformational changes in the Ca^2+^ receptors, resulting in cell apoptosis and inhibited bone resorption [38]. Chattopadhyay et al. demonstrated that Sr^2+^ could bind to Ca^2+^-sensing receptors and act as an agonist to activate several cellular responses that increase the expression of rat osteoblastic genes and induce osteoblast proliferation [39]. Furthermore, Sr^2+^ has been used as a surrogate for Ca^2+^ remedies to treat bone disorders and prevent bone fractures. Sr-ranelate (SrR) is widely used as an effective treatment for osteoporosis and found to enhance bone formation and increase overall bone quality and mineral density [40]. Despite all these advantages, prolonged oral administration of SrR was found to increase the risk of a cardiovascular infarction, and many studies proved that local administration of Sr^2+^ is more beneficial [41,42,43]. Recently, with the emergence of BTE and biomaterials in the treatment of bone diseases, Sr-enriched biomaterials showed an advantageous effect that allowed local Sr delivery, reducing the side effects [44]. Sr^2+^ has been introduced with Ca^2+^ phosphate biomaterials due to their resemblance to natural bone and the easiness of Sr incorporation into their structure [45]. Additionally, a Sr-HA scaffold showed higher ALP activity in vitro compared to pure HA, while in vivo animal implantation enhanced osteogenesis [46].

Polymeric nanoparticles that possess antimicrobial properties such as silver, zirconia, zinc oxide, etc., have been introduced to medical devices in the field of dental prosthetics and periodontal diseases. However, further in vivo experiments are needed to assess their biocompatibility, cytotoxicity, and degradation [47]. In addition, polymer matrices that contain silver nanoparticles proved to be effective in wound dressing and prevented bacterial growth at the wound site; however, these particles tend to agglomerate with time which reduces their antimicrobial effect [48].

Poly (lactic-co-glycolic acid) (PLGA) is an FDA-approved polymer that is biocompatible and has controllable mechanical properties and degradation rate. PLGA is widely used as a delivery vehicle for drugs or bioactive factors [49]. However, its use as a scaffold in BTE has been limited due to its low osteoinductivity. As a result, PLGA is always used in a composite accompanied by other biomaterials [50]. Studies have shown that the incorporation of HA into a PLGA polymer can enhance osteogenic cell proliferation and differentiation, while an in vivo HA-PLGA scaffold exhibited good structural stability and mechanical properties [51,52]. Furthermore, some studies used PLGA to encapsulate antibacterial agents which significantly reduced bacterial growth [53,54,55], but this could increase the burden of antibiotic-resistant bacterial species, and also raise the concern of a burst release of the encapsulated antibiotic [56]. Silver and copper ions (Ag^+^ and Cu^2+^) have also been extensively studied due to their antimicrobial properties [57]. Ag^+^ was found to have a high cytotoxic effect compared to Cu^2+^, while Cu^2+^’s antibacterial effect remained only for a short time frame [58].

This study aimed to fabricate a multipurpose bio-composite bone scaffold of Sr/Zn doped nHAp-PLGA that has antibacterial activity and is cell friendly. Zn^2+^ and Sr^2+^ were chosen to dope nHAp in this study as Zn^2+^ has a dual effect of enhancing bone regeneration and reducing bacterial growth, while Sr^2+^ can enhance osteoblastic gene expression and increase bone density. Meanwhile, PLGA was added to increase the scaffold stability and mechanical properties. 

In our previously published work [59], we analyzed the influence of Sr^2+^ and Zn^2+^ doping on nHAp crystallinity, and we were successfully able to synthesize Sr/Zn-doped nHAp-PLGA composite scaffolds with adequate porosity, bioactivity, and degradability. The XRD pattern and FTIR spectra revealed the phase composition and crystal properties of nHAp in both Sr/Zn-doped powders and composite scaffolds and also confirmed the incorporation of PLGA in the scaffolds. Crystallinity decreased while the Sr^2+^/Zn^2+^ concentration increased, and composite scaffolds with less crystalline nHAp produced a bioactive layer that was suitable for bone regeneration. The scaffolds were able to form an orthophosphate layer on the surface when immersed in simulated body fluid, which was confirmed by SEM and TEM studies [59]. Composite scaffolds with PLGA and 4% Sr/Zn-nHAp exhibited the most abundant crystal growth after 2 weeks of submersion in SBF [59]. The porous structure of the scaffolds was confirmed in the SEM images. The composite scaffolds showed interconnected, widely distributed pores. The average pore size for PLGA-nHAp, PLGA-2.5% Sr/Zn-nHAp, and PLGA-4% Sr/Zn-nHAp scaffolds ranged between 189 ± 10.26 to 406 ± 26.54 (mean ± SEM). The results suggested a statistically significant increase (*p* < 0.0001) in the pore size of the composite scaffolds when we doped them with strontium and zinc ions [59]. In addition, PLGA incorporation proved to reinforce the mechanical properties and scaffolds were able to release Sr and Zn ions in vitro for up to three weeks [59].

In the present work, we investigated the antimicrobial properties of Zn /Srdoped nHAp with and without PLGA polymer, and assessed the effect of PLGA incorporation on Zn^2+^ antimicrobial activity. Furthermore, this work examined cytocompatibility and osteoblastic cell proliferation on Zn/Sr-nHAp-PLGA composite scaffolds. The study also optimized the concentration of doping elements (Zn/Sr) and measured their release in simulated body fluid (SBF) using ICP-MS, making sure that the levels of these ions were within the normal range that could enhance cell viability, hence augmenting bone formation.

## 2. Methods

### 2.1. Preparation of Nano-Hydroxyapatite

nHAp was prepared using the chemical precipitation method [60,61]. Briefly, 0.06 M ammonium phosphate dibasic [(NH_4_)_2_HPO_4_, 98% Sigma-Aldrich (Saint Louis, MO, USA)] and 0.1 M calcium nitrate tetrahydrate [Ca(NO_3_)_2_.4H_2_O, 99% Sigma-Aldrich] solutions were prepared separately and dissolved in deionized water. After that, the phosphate-containing solution was added dropwise to the calcium-containing solution and the Ca/P ratio was kept at 1.67. The pH was adjusted to 11–12 using 10 N sodium hydroxide [NaOH, 98% Sigma-Aldrich] and the resulting solutions were kept under stirring conditions for 1 h. The mixture was then aged overnight, and the white precipitate was filtered and washed with distilled water 3 times. The final slurry was dried in an oven at 80 °C for 24 h and calcinated at 300 °C for 1 h.

### 2.2. Preparation of Zn/Sr-Substituted Nano-Hydroxyapatite

A phosphate-containing solution and a calcium-containing solution were prepared as previously mentioned. Zn in the form of zinc nitrate hexahydrate [Zn (NO_3_)_2_.6H_2_O, 98% Daejung Chemicals & Metals Co., Ltd.] and/or strontium nitrate [Sr (NO_3_)_2_, 99% Merck, Darmstadt, Germany] was added to the Ca-containing solution and the concentration of Zn and/or Sr was set at 1%, 2.5%, and 4%. Then, the phosphorous (P)-containing solution was added dropwise to the Ca and Zn/Sr solution, and the pH was adjusted to 11–12. After that, the mixture was stirred at 100 °C for 1 h and aged overnight. The resulting white precipitate was filtered and washed 3 times with distilled water then dried in an oven at 80 °C for 24 h, followed by calcination at 300 °C for 1 h [62].

### 2.3. Preparation of Zn/Sr-nHAp-PLGA

The composite scaffolds were prepared according to [63] with some modifications. Briefly, different ratios of Zn/Sr-nHAp (see Table 1) and PLGA polymer [lactide: glycolide (75:25), mol wt. 66,000–107,000, Sigma Aldrich] were dissolved separately in organic solvent dichloromethane (DCM, Merck) and vortexed at 1000 RPM for 15 min. Zn/Sr-nHAp dispersion was added dropwise to the PLGA/DCM solution and the resulting mixture was vortexed at 2000 RPM for 30 min, and then kept in the oven at 70 °C for 15 min to evaporate the solvent. The resulting slurry was washed with ethanol 3 times to remove the DCM residues and left in the oven at 50 °C for 48 h to dry, followed by calcination at 150 °C for 4 h.

### 2.4. Scaffold Fabrication

**Figure 1:**
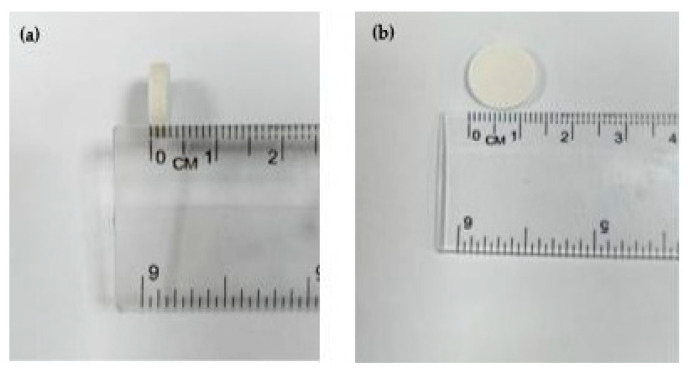
Fabricated scaffolds’ (**a**) side view and (**b**) top view.

From each prepared powder, 500 mg was compressed using a hydraulic pellet press at 5000–10,000 psi for 1 min to form a disc shape of 13 × 3 (W × H) mm (Figure 1). Disc scaffolds were subjected to supercritical CO_2_, as in our previously published study [59]. Before each experiment, discs were placed on 24-well plates and sterilized using a UV light for 30 min on each side.

### 2.5. Assessment of Scaffolds’ Antibacterial Activity

Scaffold antibacterial activity was measured against *Staphylococcus aureus* [ATCC (25923), Microbiologics, St Cloud, MN, USA] by counting bacterial colonies on tryptone soya agar (TSA, Mast, Bootle, UK)) after direct contact with the scaffold, as described by Resmim et al. and Ofudje et al. [62,64]. Briefly, the bacterial density was adjusted to 5 × 10^6^ CFU/mL using a McFarland densitometer and tryptone soya broth. A total of 2 mL of the adjusted bacterial suspension was added into each scaffold in wells; a well without scaffold was used as a growth-positive control (G.C), and plates were incubated at 37 °C for 24 h. After incubation, the bacterial suspension was aspirated, 1 mL of phosphate-buffered saline (PBS) was added to each scaffold, and the plates were placed in a shaker at 100 RPM for 15 min to remove scaffold-attached bacteria [64]. Thereafter, 100 µL of the test solution (bacteria in PBS) was retrieved and serially diluted to 10^−8^. The agar plates were then divided into 8 equal square sectors and 5µL of the appropriate dilution was dropped onto the agar surface of each sector and left upright to spread and dry for 15–20 min. Agar plates were then inverted and incubated at 37 °C for 24 h for colony formation. The final CFU/mL in the original sample was calculated according to the equation: CFU/mL = the average number of colonies for a dilution × total dilutions of the sample. Furthermore, the percentage of antibacterial properties of different scaffolds was calculated using the formula: antibacterial rate % = (CFU _control_ − CFU _test_)/CFU _control_ × 100% [65]. All trials were performed in triplicate and the results were normalized by calculation of the arithmetic average.

### 2.6. Cell Culture

**Figure 2:**
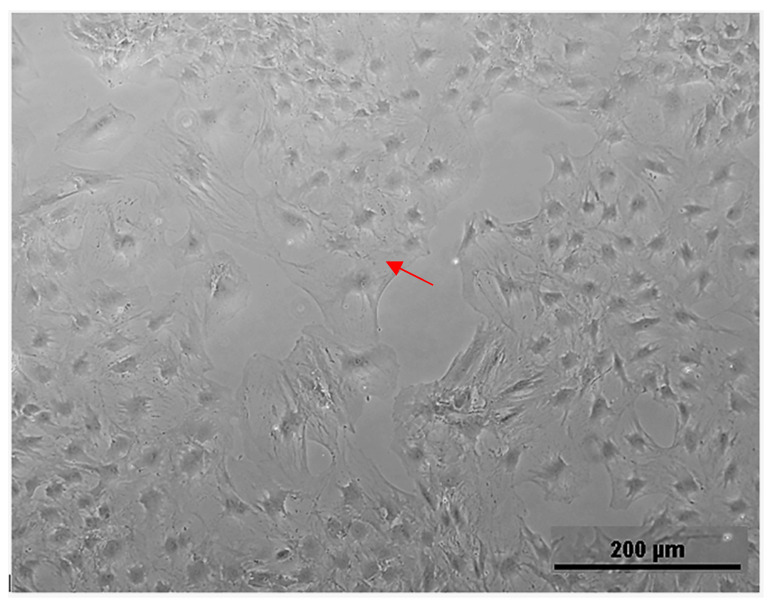
Cultured ROb cells showing their characteristic stellate-to-spindle-shaped appearance, indicated with a red arrow (Scale bar = 200 µm).

In this study, primary rat osteoblasts’ cell line (ROb) was purchased from (Cell Applications Inc., San Diego, CA, USA) and all the tissue culture reagents were purchased from Sigma-Aldrich. Cells were cultured using standard protocols, as per company instructions, following the standard sterilization technique and safety rules. ROb cells were cultured in a rat osteoblast growth medium supplemented with 10% FBS and 1% penicillin-streptomycin antibiotic and incubated in a 5% CO_2_ incubator at 37 °C. Cells were passaged when reaching 80–90% confluency (Figure 2). The medium was refreshed every 2–3 days and passages number 4–7 were used for scaffold seeding in this experiment. Cells in the culture flask were observed using an inverted Olympus microscope IX70 with a digital camera DP70.

### 2.7. Scaffold Seeding

The fabricated scaffolds used to check cell viability were nHAp, PLGA-nHAp, 1% Sr/Zn-nHAp-PLGA, 2.5% Sr/Zn-nHAp-PLGA, and 4% Sr/Zn-nHAp-PLGA, and *n* = 3 for each scaffold. Prior to cell seeding, the scaffolds were placed aseptically in 24-well plates and sterilized using a UV light for 30 min on each side, then soaked in 1 mL of complete culture medium for 1 h. Cells were trypsinized and the seeding density was adjusted to 1 × 10^5^ cells per scaffold. A total of 35 µL of the adjusted cells suspension was added dropwise to each scaffold, followed by incubation at 37 °C in 5% CO_2_ for 1 h to allow cell attachment. After that, 2 mL of complete culture medium was added to each scaffold, and the plates were returned to the incubator. The culture medium was changed every 2–3 days [66].

### 2.8. MTT Assay

On day 2 and day 7, osteoblast cell proliferation was assessed using a 3-(4,5-dimethyl thiazolyl-2)-2,5-diphenyl-tetrazolium bromide (MTT) assay kit (Abcam, ab211091, Boston, MA, USA). In this assay, living cells cleave MTT-soluble tetrazolium salts and convert them into insoluble purple formazan, which is then solubilized, and the resulting color intensity is directly proportional to the number of living cells [67]. Briefly, the medium was aspirated from the scaffolds, 1 mL of MTT reagent was added to each scaffold, and then plates were incubated at 37 °C for 4 h. After incubation, the MTT reagent was removed and 1 mL of MTT solvent was added to each well. Subsequently, plates were wrapped with aluminum foil and agitated on an orbital shaker for 15 min. The test solution was transferred into a new 24-well plate and the optical density (OD) was measured at 590 nm using a Tecan infinite M200 PRO microplate reader [68].

### 2.9. Sr and Zn Ion Release Study

The ion release study was performed according to our previously published work [59]. Briefly, each scaffold was immersed in SBF at 37 °C for 28 days. The scaffolds were removed at different time intervals and the Sr and Zn ions’ release in SBF fluid was measured using inductively coupled plasma mass spectrometry (ICP-MS; NexION 300X, PerkinElmer, Waltham, MA, USA).

### 2.10. Statistical Analysis

All experiments were measured in triplicate and data were analyzed using SPSS software (version 28.0) using one-way analysis of variance (ANOVA) and post hoc for multiple comparisons. Differences were considered statistically significant when the *p*-value was <0.05.

## 3. Results and Discussion

### 3.1. Scaffolds Antibacterial Activity

**Figure 3:**
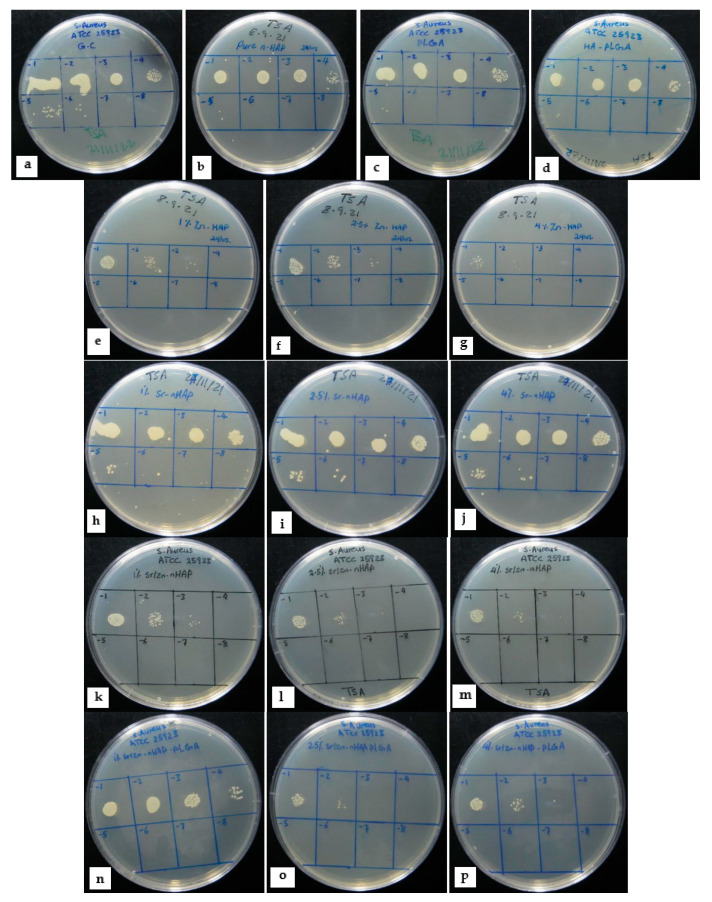
Photographs of bacterial colonies in TSA plates after 24 h of direct contact with different scaffolds. Each square sector represents a serial dilution from 10^−1^ to 10^−8^, left to right. (**a**) G.C, (**b**) pure nHAp, (**c**) Pure PLGA, (**d**) nHAp-PLGA, (**e**) 1% Zn-nHAp, (**f**) 2.5% Zn-nHAp, (**g**) 4% Zn-nHAp, (**h**) 1% Sr-nHAp, (**i**) 2.5% Sr-nHAp, (**j**) 4% Sr-nHAp, (**k**) 1% Sr/Zn-nHAp, (**l**) 2.5% Sr/Zn-nHAp, (**m**) 4% Sr/Zn-nHAp, (**n**) 1% Sr/Zn-nHAp-PLGA, (**o**) 2.5% Sr/Zn-nHAp-PLGA, (**p**) 4% Sr/Zn-nHAp-PLGA.

Our focus in this study was to assess the antibacterial property of the fabricated scaffolds [nHAp, PLGA, nHAp-PLGA, Zn-nHAp, Sr-nHAp, Sr/Zn-nHAp, and Sr/Zn-nHAp-PLGA] with different doping percentages of Sr and Zn ions (1%, 2.5%, and 4%). Figure 3 represents colony forming units (CFU) of *S. aureus* in TSA after 24 h of direct contact with the different scaffolds. Figure 3a–d demonstrates bacterial CFU in the growth control (G.C), i.e., growth without any scaffold, nHAp, PLGA, and PLGA-nHAp, which showed a relatively similar bacterial number. Moreover, in the Zn-nHAp scaffolds in Figure 3e–g, all Zn concentrations showed a decrease in CFU numbers, the reduction was dose-dependent, and the scaffolds with the highest Zn^2+^ concentration (4% Zn-nHAp) showed the lowest CFU numbers; hence, they had the best antibacterial activity. Sr-nHAp scaffolds did not show any antibacterial activity in Figure 3h–j and the CFU number was similarly close to G.C and nHAp. Sr/Zn nHAp with and without PLGA in Figure 3k–p showed a reduction in CFU number as the Zn^2+^ concentration increased, and a 4% doping percentage attained the maximum inhibitory effect for both scaffolds with and without PLGA.

**Figure 4:**
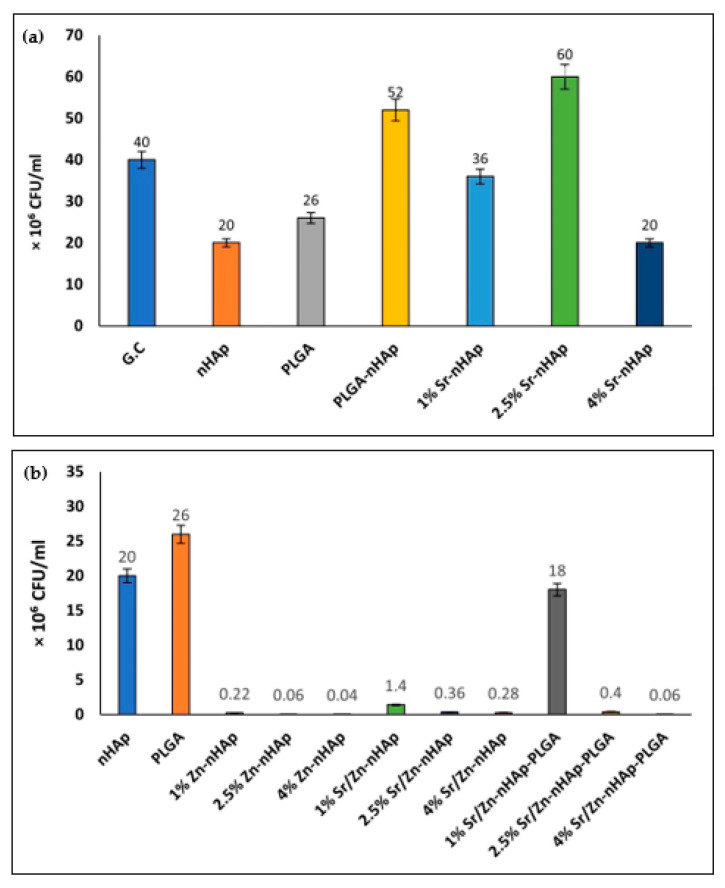
Bacterial CFU counts after 24 h of incubation with different scaffolds (**a**) G.C, nHAp, PLGA, PLGA- nHAp, 1% Sr-nHAp, 2.5% Sr-nHAp, 4% Sr-nHAp; (**b**) 1% Zn-nHAp, 2.5% Zn-nHAp, 4% Zn-nHAp, 1% Sr/Zn-nHAp, 2.5% Sr/Zn-nHAp, 4% Sr/Zn-nHAp, 1% Zn/Sr-nHAp-PLGA, 2.5% Zn/Sr-nHAp-PLGA and 4% Zn/Sr-nHAp-PLGA.

Figure 4a,b shows the exact numbers of bacteria CFU. The results showed that the bacterial number in G.C was 4 × 10^7^ CFU/mL; in nHAp, it was 2 × 10^7^ CFU/mL, while in PLGA, the CFU count was 2.6 × 10^7^ CFU/mL. A drop in the CFU number was observed in all Zn^2+^-containing scaffolds, with 4% Zn showing the lowest CFU number of 28 × 10^4^ CFU/mL in 4% Sr/Zn-nHAp scaffolds, and 6 × 10^4^ CFU/mL in 4% Sr/Zn-nHAp-PLGA, indicating that PLGA incorporation into the scaffolds did not affect Zn^2+^’s antibacterial action.

The bacterial growth inhibition percentage of the different scaffolds was calculated from CFU numbers, using the nHAp scaffold as a control (Table 2). Zn^2+^-containing scaffolds showed the best growth inhibition percentage that ranged between 98.6 ± 0.2 and 99.7 ± 0.1% in 4% Zn concentrations. Sr^2+^-containing scaffolds in 1%, 2.5%, and 4% Sr-nHAp did not show any bacterial inhibitory effect unless accompanied with Zn^2+^, which is also in agreement with other studies where Sr-HA did not reveal any antibacterial properties unless accompanied by other metals such as Ag^+^ (silver) and Se^4+^ (selenium), as they are known to exhibit antibacterial effect [69,70,71].

**Figure 5:**
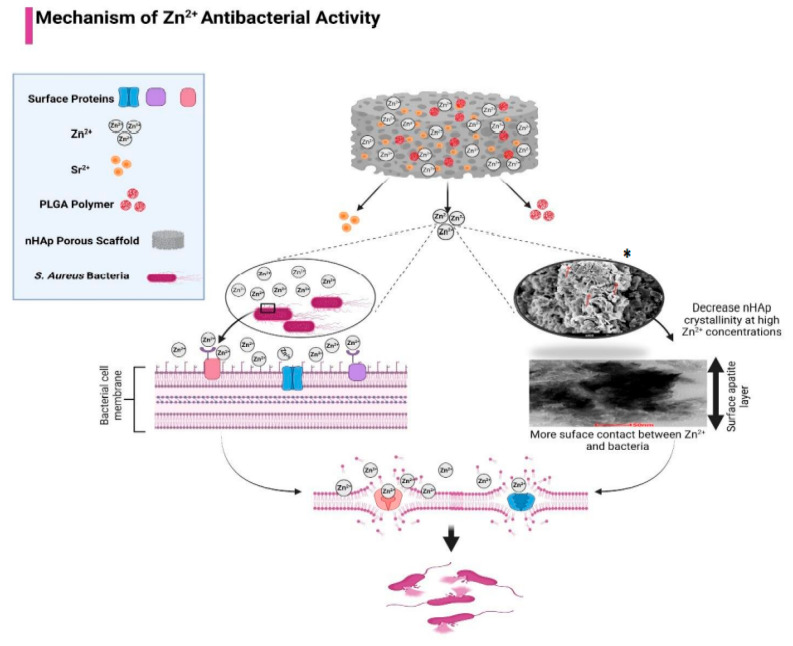
Mechanism of Zn^2+^ antibacterial effect. Created with BioRender.com. * [59] Hassan et al.

Several previous studies have reported the antimicrobial properties of Zn-nHAp against *Staphylococcus aureus* and *Escherichia coli* [25,57,62,72,73,74]. Multiple factors could contribute to this bactericidal effect. One of them is the fact that Zn ions can bind to some structural proteins in the bacterial cell membrane, altering the membrane permeability and killing the bacteria [62,75]. Additionally, the decrease in Zn-nHAp crystallinity in the higher Zn^2+^ concentrations will enhance the growth of the apatite layer (Figure 5) and, consequently, increase the surface area which will facilitate the contact between Zn ions and the bacterial cell membrane, resulting in more bacterial death [72]. This explains the dose-dependent reduction in bacterial CFU numbers as the Zn^2+^ concentration increased. The enhanced antibacterial effect in higher Zn^2+^ concentrations was also observed by Ofudje et al. and Valarmathi et al. [62,76]. The PLGA polymer has been extensively studied as a drug carrier due to its many beneficial properties [77,78,79]. In many studies, PLGA was used to encapsulate antibiotic agents, whereas, in some studies, PLGA was used as a composite in bone scaffolds. In our previous study, PLGA incorporation into nHAp showed an increase in the scaffold mechanical properties [59], while in this study, PLGA was found to have no impact or a weakening effect on Zn^2+^ antibacterial activity, which makes it a suitable candidate to use in antimicrobial bone scaffolds in combination with Sr/Zn-doped nHAp.

### 3.2. Cell Proliferation Using MTT Assay

**Figure 6:**
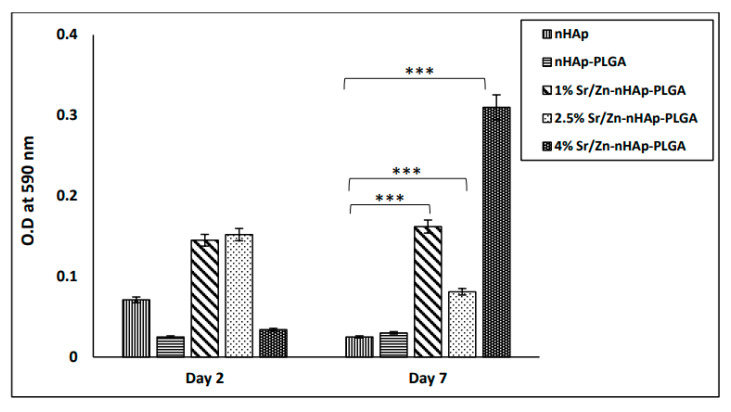
MTT assay for osteoblasts cultured for 2 and 7 days on different scaffolds: (nHAp, nHAp-PLGA, 1% Zn/Sr-nHAp-PLGA, 2.5% Zn/Sr-nHAp-PLGA, and 4% Zn/Sr-nHAp-PLGA). *** *p* ≤ 0.001 compared to nHAp.

MTT assay was performed to assess osteoblast cell proliferation in the different fabricated scaffolds to evaluate their cytocompatibility. Many previous studies confirmed nHAp cytocompatibility and its favorable effect on osteoblast proliferation and differentiation [80,81,82]. Therefore, cell proliferation on nHAp was used as a control to evaluate the Sr/Zn doping effect. MTT assay (Figure 6) showed that the number of metabolically active cells on day 2 in nHAp and nHAp-PLGA scaffolds was less when compared to Sr and Zn ion-doped scaffolds; however, the difference was not statistically significant. On day 7, the optical densities of cells in scaffolds devoid of Sr/Zn ions were slightly decreased and cells were not able to maintain their numbers, while all scaffolds co-doped with Sr/Zn ions showed a significant increase in the cell numbers compared to pure nHAp (*p* < 0.001). The highest concentration of Sr/Zn ions as in the 4% example were able to maintain the number of viable cells for 7 days and showed the greatest number of viable cells. These results suggest that 4% Sr/Zn-nHAp-PLGA scaffolds could be preferable for cell proliferation and viability. Wang et al. also demonstrated that BMSCs cultured in Sr/Zn co-doped HA scaffolds for 7 days, showed a stronger proliferative ability and ALP activity compared to single-ion-doped and pure HA scaffolds [83].

The mechanism by which Zn and Sr ions can affect osteoblast behavior was investigated previously. Zn and Sr ions can stimulate osteogenesis by interfering with several signaling pathways that can regulate gene expression, proliferation, differentiation, and collagen matrix mineralization in cultured osteoblasts [84,85]. Additionally, an earlier study showed that zinc-containing nanoparticles loaded to cellulose and chitosan-based hydrogels promoted vascular endothelial growth factor (VEGF) expression and angiogenesis, which is useful for cell proliferation and tissue regeneration [86].

The optimal effective dosage for Zn or Sr ions that can accelerate cell proliferation without being toxic to the cells is debatable. We found the literature to be contradictory on zinc concentration; some studies stated that 5% and up to 6% Zn ion concentration can enhance cell proliferation while increasing the Zn^2+^ concentration to 10% and 20% resulted in a significant drop in cell numbers [87,88,89]. Additionally, Fernandes et al. demonstrated that 5% Zn-HA powder improved the osteogenesis of rat calvarial bone defects almost as much as autografts, and demonstrated a slower degradation rate compared to pure HA and autografts [90]. HA with 13% Zn^2+^ contents can lead to Mg ion depletion and replacement with Zn ions, which will jeopardize cell survival, as Mg ions are normally found to bind to some structural proteins that help to maintain cell membrane integrity and are involved in several vital processes such as DNA synthesis and polymerase activity [74]. An increase in ROS formation which may induce oxidative stress and lead to cell damage is also reported with the use of higher concentrations of Zn^2+^. [74]. On the other hand, Popa et al. and Ullah et al. reported no cytotoxic effect at a 10% Zn concentration [91,92]. X-ray diffraction (XRD) and Fourier-transform infrared spectroscopy (FTIR) studies proved that increasing Zn^2+^ concentrations to more than 12.3 wt.% can weaken the HA structure due to the loss of the apatite phase and replacement with a non-apatite phase of Zn-β tricalcium phosphate (β-TCP), as more Ca ions will be substituted by Zn ions, resulting in different solubility and ion release rates by the scaffold [93]. On the other hand, several studies did not show a cytotoxic effect for 10% Sr-HA or reported weak cytotoxicity [94,95], suggesting that cells can tolerate high Sr^2+^ concentrations in contradistinction to Zn^2+^. This study investigated 1%, 2.5%, and 4% of Zn^2+^ and Sr^2+^ doped to nHAp. According to our results, a 4% Zn^2+^ and Sr^2+^ doping percentage maximized the scaffold antibacterial effect and enhanced osteoblastic cell survival and proliferation. In addition, PLGA incorporation into the scaffold did not jeopardize Zn^2+^ antibacterial action and enhanced scaffold cytocompatibility and mechanical properties.

### 3.3. Sr and Zn Ion Release Using ICP-MS

**Figure 7:**
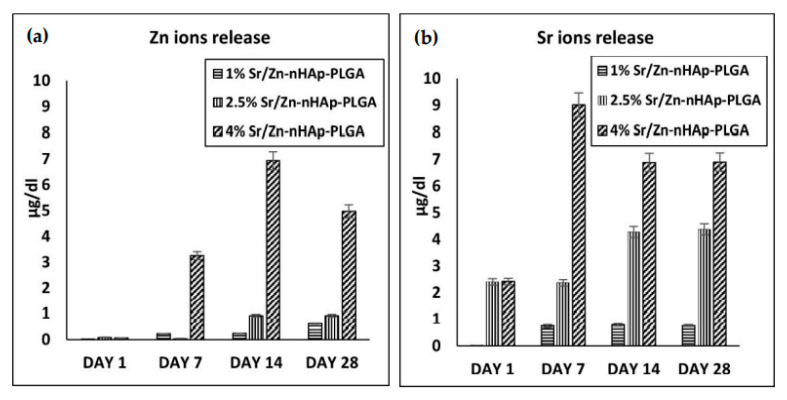
Ion release profile using ICP-MS for 1% Zn/Sr-nHAp-PLGA, 2.5% Zn/Sr-nHAp-PLGA, and 4% Zn/Sr-nHAp-PLGA. (**a**) Zinc ions; (**b**) strontium ions.

The ions release profile of Sr/Zn-nHAp-PLGA scaffolds was measured using inductive coupled plasma-mass spectrometry ICP-MS on days 1, 7, 14, and 28 after immersion in SBF. The results in Figure 7a,b show that the release profile started on day 1 and was maximized in 4% Sr/Zn-nHAp-PLGA scaffolds for all days. This will be helpful for bone healing as ions will be readily available as early as the first week. Zn and Sr ion release peaked at day 14 for Zn ions and day 7 for Sr ions.

A key factor that needs to be under control is the amount of Zn/Sr ions released into the surrounding medium as the scaffold degrades, which will be taken up by cells and affect their viability. The reference range for Zn^2+^ levels in human serum is 60–120 µg/dL, while for Sr^2+^, it is 1.9–9.6 µg/dL [96,97]. The highest release profile for Zn and Sr ions was 6.9 µg/dL and 9.02 µg/dL, respectively. These levels did not exceed the normal concentration range; hence, we can consider 4% Zn/Sr-nHAp-PLGA as a safe and cytocompatible scaffold according to in vitro studies. In our study, we tried to limit Zn^2+^ concentrations to 4%, as higher concentrations may result in a higher release profile that could outrange the normal levels and may increase the risk of cytotoxicity. Scaffold implantation in an animal model is needed further to confirm in vivo biocompatibility and bone regeneration ability.

Designing an ideal bone scaffold with appropriate porosity, biocompatibility, and tunable mechanical properties is still a challenge. Natural polymers such as chitosan, alginate, silk, and collagen demonstrate good biocompatibility, degradability, and bioactivity. However, they have poor mechanical properties. As a result, they are usually used in the form of blends of two or three components to improve their mechanical properties [98,99]. In addition, the functionalization of these polymers is difficult, which reduces their bone regeneration ability [11]. The composite scaffold proposed in this study is composed mainly of a natural bone component (nHAp) and doped with Sr^2+^ and Zn^2+^ metal ions, which are also a natural constituent of bone that can introduce a dual effect of enhancing bone regeneration and an antibacterial effect to the scaffold. Moreover, the scaffolds’ mechanical properties were improved by the addition of the PLGA polymer to the scaffold, as shown in our previous work [59].

## 4. Conclusions

Introducing an antibacterial function to the scaffold without compromising scaffold biocompatibility is substantial. The scaffolds impregnated with antibiotics may increase the burden of antibiotic resistance species and increase the concern of burst release of these drugs, which will leave the scaffold ineffective for bacterial killing. The synthesis of scaffolds with surface coats that inhibit bacterial adhesion and growth such as nanopillars needs an extreme synthesis environment which can affect the polymer’s properties. Scaffolds doped with metal ions such as Ag^+^ were found to have a high cytotoxic effect, while Cu^2+^’s antibacterial effect remained only for a short time. In comparison, Zn^2+^ has good antibacterial activity at certain limited concentrations, while it was also found to enhance osteoblast gene expression and increase bone mineralization [33].

In this study, we presented a novel Sr/Zn co-doped nHAp-PLGA scaffold with an antibacterial effect against *S. aureus* that reached 99.7% bacterial growth inhibition in the 4% Sr/Zn-nHAp-PLGA composite scaffold; hence, this was the best dosage required. This antibacterial activity increased with increasing Zn ion concentrations, while PLGA polymer incorporation into the scaffold did not affect this antibacterial activity. At the same time, it enhanced the scaffold’s mechanical property and stability, as shown in our earlier study [59]. MTT assay showed that Zn/Sr co-doped nHAp-PLGA scaffolds provided a friendly environment for osteoblast cell proliferation compared to pure nHAp. Our results suggest that 4% Sr/Zn-nHAp-PLGA is a promising candidate for bone tissue engineering applications with excellent antimicrobial activity and cytocompatibility.

## Figures and Tables

**Table 1:** Composition of different Sr/Zn-nHAp-PLGA scaffolds.

Scaffolds	Sr (mol%)	Zn (mol%)	nHAp (mol%)	PLGA (mol%)
nHAp	0	0	1	0
PLGA-nHAp	0	0	1	3
1% Sr/Zn-nHAp-PLGA	1	1	1	3
2.5% Sr/Zn-nHAP-PLGA	2.5	2.5	1	3
4% Sr/Zn-nHAp-PLGA	4	4	1	3

**Table 2:** Bacterial growth inhibition percentage for different scaffolds ± SEM.

Composite Scaffolds	Growth Inhibition %
Pure nHAp	0%
PLGA	0%
PLGA-nHAp	0%
1%, 2.5%, 4% Sr-nHAp	0%
1% Zn-nHAp	98.9 ± 0.5%
2.5% Zn-nHAp	99.7 ± 1.65%
4% Zn-nHAp	99.8 ± 0.1%
1% Sr/Zn-nHAp	93 ± 3.4%
2.5% Sr/Zn-nHAp	98.2 ± 0.4%
4% Sr/Zn-nHAp	98.6 ± 0.2%
1% Zn/Sr-nHAp-PLGA	10 ± 4.5%
2.5% Zn/Sr-nHAp-PLGA	98 ± 0.57%
4% Zn/Sr-nHAp-PLGA	99.7 ± 0.1%

## Data Availability

The data presented in this study are available on request from the corresponding author.
